# Mechanisms of Microstructural and Defect Evolution in Laser Powder Bed Fusion-Fabricated In625 Induced by Heat Treatment

**DOI:** 10.3390/ma19091713

**Published:** 2026-04-23

**Authors:** Qing Chen, Yi Liu, Xuxing Duan, Xianjun Zhang, Gening He, Yu Sun, Changyuan Li

**Affiliations:** 1 National Key Laboratory of Nuclear Reactor Technology, Nuclear Power Institute of China, Chengdu 610213, China; chqing84@163.com (Q.C.); es803@npic.ac.cn (X.D.); keban@npic.ac.cn (X.Z.); hegening@npic.ac.cn (G.H.); sy954961542@163.com (Y.S.); cfar2333@163.com (C.L.); 2 State Key Laboratory of Advanced Nuclear Energy Technology, Nuclear Power Institute of China, Chengdu 610213, China; 3 Nuclear Power Additive Manufacturing Key Laboratory of Sichuan Province, Nuclear Power Institute of China, Chengdu 610213, China; 4 School of Energy and Power Engineering, Xi’an Jiaotong University, Xi’an 710049, China

**Keywords:** laser powder bed fusion, heat treatment, In625, microstructure, defects

## Abstract

**Highlights:**

- Optimized L-PBF parameters enabled high-density fabrication of In625 with stable melt-pool morphology.
- Solution heat treatment promoted grain equiaxation and reduced lattice distortion in L-PBF In625.
- Porosity exhibited a non-monotonic evolution, decreasing at 1090 °C and increasing at 1150 °C.
- Heat treatment at 1150 °C provided the best overall balance among strength, ductility, and anisotropy reduction.
- The results clarify the microstructure–defect–property relationship in heat-treated L-PBF In625.

**What are the main findings?**

- Optimized L-PBF parameters enabled the fabrication of high-density In625 components.
- Heat treatment temperature strongly affects porosity and grain evolution.
- Grain equiaxation occurs below 1090 °C in L-PBF-fabricated In625.

**What are the implications of the main findings?**

- Heat treatment enables simultaneous optimization of strength and ductility.
- Defect evolution explains the reduction in anisotropy after heat treatment.
- The results guide engineering applications of L-PBF In625.

**Abstract:**

Heat treatment is essential for In625 fabricated by laser powder bed fusion (L-PBF), as it significantly influences microstructural evolution, defect behavior, and mechanical performance. In this study, the effects of different solution heat treatments on L-PBF-fabricated In625 were systematically investigated. Industrial computed tomography was employed to characterize internal defects before and after heat treatment, while optical microscopy, EBSD, TEM, and EDS were used to analyze microstructural evolution. Room-temperature tensile tests evaluated mechanical properties. The results show that heat treatment at 1090 °C reduces porosity from 0.33% to 0.25%, whereas increasing the temperature to 1150 °C results in a further increase in porosity to 0.45%. This non-monotonic behavior is interpreted as the result of competing mechanisms, including partial closure of small pores at 1090 °C and pore coarsening/enlargement at higher temperatures, with the latter possibly involving the growth of sub-resolution pores into the CT-detectable range. Complete grain equiaxiality occurs after heat treatment at 1090 °C or higher, with average grain sizes below 100 μm, although grain coarsening becomes pronounced at higher temperatures. Samples heat-treated at 1150 °C exhibit reduced mechanical anisotropy, achieving tensile strength above 919 MPa and elongation up to 60%. These results clarify the mechanisms by which heat treatment governs microstructure–defect–property relationships in L-PBF In625, guiding its engineering application.

## 1. Introduction

Metal additive manufacturing (AM) technologies have been increasingly applied in high-end industrial fields such as nuclear energy, aerospace, and biomedical engineering due to their capability to fabricate geometrically complex components with high material utilization efficiency and design flexibility [1]. Among various AM techniques, laser powder bed fusion (L-PBF) has attracted significant attention, owing to its high forming accuracy, excellent surface quality, and near-net-shape manufacturing capability, making it particularly suitable for producing small-to-medium-sized components with intricate geometries [2,3]. However, L-PBF is characterized by extremely rapid melting and solidification, which inevitably introduce complex thermal histories, resulting in microstructural heterogeneity, residual defects, and mechanical anisotropy in as-built components.

Nickel-based superalloy In625 is a solid-solution-strengthened alloy primarily strengthened by Mo and Nb. Owing to its excellent high-temperature strength, corrosion resistance, and microstructural stability, In625 has been widely used as a critical structural material in high-temperature, high-pressure service environments, particularly in the aerospace and nuclear industries [4,5,6,7].

L-PBF offers a promising approach for manufacturing In625 components with complex geometries; however, the as-built microstructure and defect characteristics produced by L-PBF differ significantly from those of conventionally processed counterparts, which can adversely affect the mechanical performance and service reliability of the material. Post-processing heat treatment is therefore essential for L-PBF-fabricated In625 components to meet engineering application requirements. Appropriate heat treatment can effectively regulate microstructural features, reduce residual stress, promote grain equiaxation, and modify defect morphology, thereby improving the balance between strength and ductility [8,9,10]. Nevertheless, the response of L-PBF In625 to heat treatment is strongly dependent on both the as-built microstructure and the selected heat-treatment parameters. In particular, defects generated during the L-PBF process—such as lack-of-fusion pores and gas-induced micropores—may evolve differently under elevated temperatures, directly influencing the final mechanical properties after heat treatment. Although previous studies have investigated the effects of L-PBF process parameters and post-heat treatment on In625 alloys, a systematic understanding of the coupled relationships among additive manufacturing parameters, heat-treatment conditions, microstructural evolution, defect behavior, and mechanical anisotropy remains limited. In particular, the mechanisms governing defect closure or growth during solution heat treatment, as well as their interaction with grain evolution and precipitation behavior, have not been sufficiently clarified. Moreover, although industrial CT provides a powerful tool for three-dimensional defect characterization, CT-based analyses of heat-treatment-induced pore evolution in L-PBF In625 remain limited, especially with respect to the non-monotonic transition from pore shrinkage to pore growth. Therefore, a combined analysis of defect evolution, grain-structure changes, and mechanical anisotropy is still needed.

Based on the above considerations, this study systematically investigates the L-PBF processing and post-heat treatment behavior of In625 alloy. Optimal L-PBF process parameters are first identified to obtain high-density as-built specimens. Subsequently, a series of solution heat treatments at different temperatures is conducted to elucidate the mechanisms of microstructure evolution and internal defect formation. Industrial computed tomography (CT), optical microscopy, EBSD, TEM, and mechanical testing are employed to establish quantitative correlations among heat-treatment conditions, defect evolution, grain characteristics, and mechanical properties. The results provide mechanistic insights into the microstructure–defect–property relationships of L-PBF-fabricated In625 and offer practical guidance for its engineering application. Therefore, the purpose of this study was to evaluate how different solution heat-treatment temperatures affect the microstructure evolution, defect behavior, and mechanical anisotropy of L-PBF-fabricated In625, and to clarify the corresponding microstructure–defect–property relationships.

## 2. Materials and Methods

### 2.1. L-PBF Processing and As-Built Characterization

Gas-atomized In625 powder (Special Metals Corporation, New Hartford, CT, USA) was used as the raw material in this study. The chemical composition of the powder is listed in Table 1. The powder particle size ranged from 15 to 53 μm, with D10, D50, and D90 values of 21.5 μm, 34.3 μm, and 53.8 μm, respectively. These values were obtained from particle-size distribution analysis and are reported here using the D10/D50/D90 descriptors; conventional error bars were not included because repeated measurements for statistical dispersion analysis were not performed. Before fabrication, the build substrate and chamber were cleaned using anhydrous ethanol (Sigma-Aldrich, St. Louis, MO, USA) to minimize contamination. L-PBF fabrication was carried out using a medium-sized metal additive manufacturing system equipped with an Nd: YAG laser (EOS M290, EOS GmbH, Krailling, Germany), with a maximum laser power of 500 W and a laser spot diameter of 80–90 μm. The effective build volume of the system was 325 mm × 325 mm × 400 mm. High-purity argon was used as the protective atmosphere during the entire fabrication process to suppress oxidation. A 316 L stainless steel plate (Shanghai Baosteel, Shanghai, China) was employed as the substrate material.

To systematically evaluate the influence of processing parameters on forming quality, laser power and scanning speed were selected as the primary variables, yielding 20 parameter combinations, summarized in Table 2. The layer thickness and hatch spacing were fixed at 0.05 mm and 0.1 mm, respectively. Scanning speeds ranged from 800 to 1300 mm/s in increments of 100 mm/s, while laser power ranged from 150 to 350 W in increments of 20 W, resulting in a volumetric energy density of approximately 30–125 J/mm^3^. A stripe-scanning strategy was employed with a stripe width of 5 mm. Adjacent layers were rotated by 67° to mitigate texture accumulation. The laser beam was operated in a bidirectional scanning mode to fill each stripe.

Cubic specimens measuring 10 mm × 10 mm × 10 mm were fabricated for density measurement and microstructural characterization. After fabrication, all samples were removed from the substrate using electrical discharge wire cutting. The surfaces parallel to the build direction were sequentially ground using SiC papers with grit sizes of 180, 400, 600, 1200, and 2000, followed by polishing with a 1 μm diamond suspension (Buehler, Lake Bluff, IL, USA). Optical microscopy (Olympus BX53M, Olympus Corporation, Tokyo, Japan) was used for preliminary microstructural observation. The relative density of the specimens was measured using Archimedes’ method. To reveal the melt pool morphology and grain structure, the polished samples were etched with aqua regia (3 HCl:1 HNO_3_, Sigma-Aldrich, St. Louis, MO, USA) before optical observation.

### 2.2. Heat Treatment Procedures and Post-Processing Characterization

**Figure 1:**
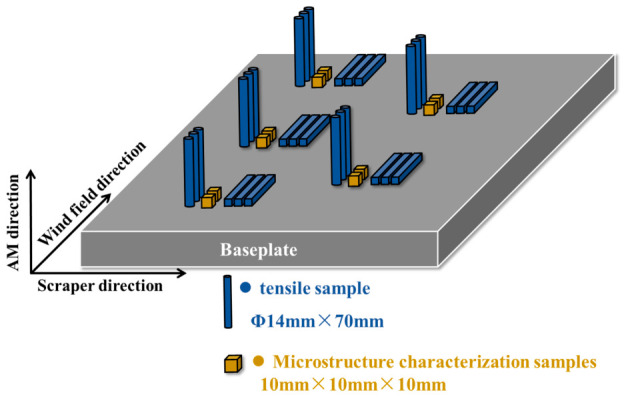
Schematic diagram of additive manufacturing specimens.

After identifying the optimal L-PBF processing parameters, additional specimens were fabricated using the selected parameter set for subsequent heat treatment investigations. The geometry, quantity, and spatial distribution of the specimens on the build plate are illustrated schematically in Figure 1. All samples were removed from the substrate by wire cutting before heat treatment.

**Figure 2:**
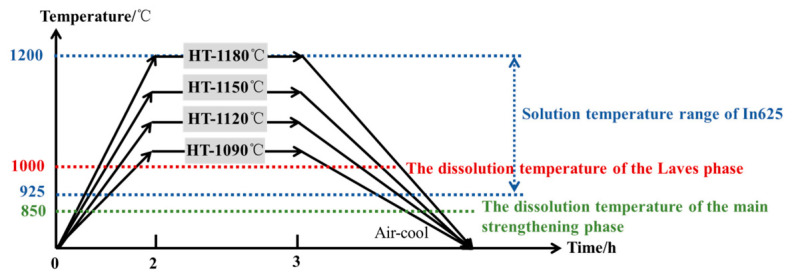
Heat treatment temperature curve.

In625 is a solid-solution-strengthened nickel-based alloy, and solution heat treatment was applied to regulate its microstructure and mechanical properties. The heat treatment temperature profile is shown in Figure 2. All specimens were heated together with the furnace to avoid oxidation during prolonged exposure at elevated temperatures, which is particularly important for large components. High-purity argon was continuously supplied throughout the heat treatment process, and the vacuum level was maintained below 5 Pa. Based on the dissolution temperature of the Laves phase (approximately 1000 °C) [11] and the equiaxial behavior of wrought In625, four solution heat-treatment temperatures were selected. The specimens were heated to 1090 °C, 1120 °C, 1150 °C, and 1180 °C, respectively, held for 1 h, and subsequently air-cooled. The heat-treated samples were designated as HT-1090 °C, HT-1120 °C, HT-1150 °C, and HT-1180 °C accordingly.

After heat treatment, comprehensive microstructural and mechanical characterizations were performed. Optical microscopy procedures were identical to those applied to as-built specimens. A field-emission scanning electron microscope (FEI Quanta 650 FEG, Thermo Fisher Scientific, Hillsboro, OR, USA) equipped with an electron backscatter diffraction (EBSD) system was used to analyze grain morphology, orientation distribution, and local misorientation. EBSD data were post-processed using Aztec Crystal software (version 4.1, Oxford Instruments, Oxford, UK). Grain-size statistics were obtained from EBSD-based grain reconstruction using Aztec Crystal software. Elemental distribution within the specimens was examined using energy-dispersive X-ray spectroscopy (EDAX, Mahwah, NJ, USA) (EDS). Internal defects were characterized using a Phoenix v|tome|x m (GE Measurement & Control Solutions, Wunstorf, Germany) multifunctional microfocus X-ray CT system with a voxel resolution of 1 μm. It should be noted that pores smaller than the voxel resolution could not be directly resolved; therefore, the apparent increase in porosity after high-temperature heat treatment may partly reflect the coarsening of previously undetectable sub-resolution pores. To ensure sufficient spatial resolution and minimize reconstruction artifacts, the thickness of all CT specimens was limited to less than 5 mm, which is well below the recommended maximum thickness for microfocus CT systems.

**Figure 3:**
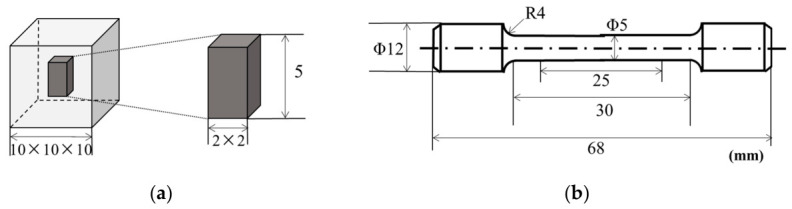
Specimen geometry for microstructural and mechanical testing. (**a**) Industrial CT test specimen; (**b**) Room-temperature tensile testing specimen.

Room-temperature tensile specimens were machined from L-PBF-fabricated cylindrical samples. Heat treatment was performed before machining to prevent dimensional distortion caused by stress release during thermal exposure. The specimen geometry is shown in Figure 3. Tensile tests were conducted using a CMT5504/5105 universal testing machine (MTS Systems Corporation, Eden Prairie, MN, USA). The specimen dimensions and testing procedures were selected according to commonly adopted metallic tensile testing practices. A constant strain rate of 0.01 mm/s was applied during testing. Specimens fabricated parallel and perpendicular to the build direction were tested to evaluate mechanical anisotropy. For each heat-treatment condition and loading orientation, multiple tensile specimens were tested, and the reported values represent the average response. At least three tensile specimens were tested for each condition and orientation, and the reported tensile strength and elongation values represent the mean values. Porosity and grain-size statistics were obtained from repeated measurements/representative datasets, as specified in the revised figure captions and discussion.

## 3. Results

### 3.1. Effect of L-PBF Process Parameters on Defect Formation

**Figure 4:**
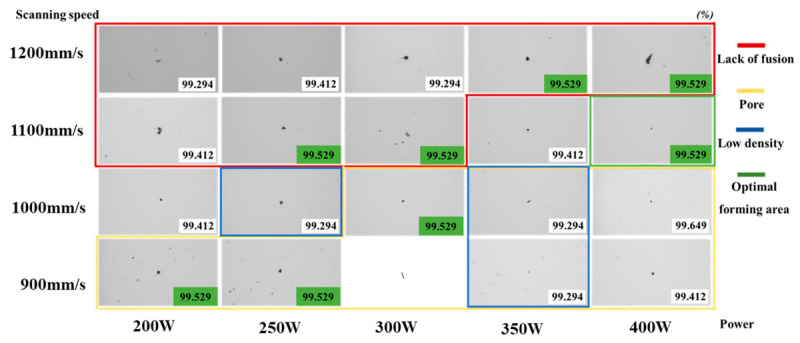
Density and optical micrograph–based defect distribution of as-deposited In625 specimens.

Figure 4 shows representative optical micrographs of typical defects in the as-deposited samples. For all samples, regions containing concentrated defects were intentionally selected for characterization to reveal the internal defect level fully. The value in the lower right corner of each image indicates the relative density of the corresponding sample. It was found that the relative density was higher for samples located along the diagonal from the lower left to the upper right of the parameter map. Statistical analysis further showed that the corresponding laser energy density in this region was relatively concentrated, ranging from 44 to 58 J/mm^3^. In addition, several samples also exhibited relatively high density, corresponding to a laser energy density range of 66–80 J/mm^3^. However, owing to the unique defect formation characteristics of laser powder bed fusion, micron-sized pores have only a limited effect on relative density. As a result, relative density alone cannot fully evaluate the forming quality of the samples, and further screening of the optimal process parameters should therefore be combined with microstructural analysis. In addition to relative density, defect size, morphology, spatial distribution, and melt-pool stability should also be considered when evaluating the forming quality of L-PBF specimens.

Defect characterization revealed that both defect type and size changed significantly under different process parameters. At low volumetric energy density (i.e., the samples located in the red region in the upper left corner of the map), lack-of-fusion defects were observed throughout the samples. This is because insufficient laser energy input could not fully melt the powder, resulting in poor bonding both between powder particles and between the powder and the previously solidified layer. At high volumetric energy density (i.e., the samples located in the yellow region in the lower right corner of the map), a large number of small pores appeared within the samples. Excessive energy input led to an excessively high melt-pool temperature, which intensified metal evaporation. Meanwhile, gas solubility in the melt pool increased with temperature; during cooling and solidification, gas solubility dropped rapidly, and gas that could not escape in time was trapped in the alloy as small pores [12,13].

At a moderate volumetric energy density (i.e., the samples located in the green region of the map), both the number and size of defects were significantly reduced. Only a few small pores, which are difficult to avoid in additive manufacturing, were observed locally. At the same time, defects such as incomplete fusion and numerous small gas pores were effectively suppressed. The corresponding optimal process parameters were a laser power of 400 W and a scanning speed of 1100 mm/s. From a more general perspective, this parameter combination corresponds to a moderate volumetric energy-density window and a relatively stable melt-pool morphology, suggesting that the optimized condition is associated not only with density maximization but also with balanced heat input and defect suppression.

### 3.2. Effect of L-PBF Process Parameters on Microstructure

**Figure 5:**
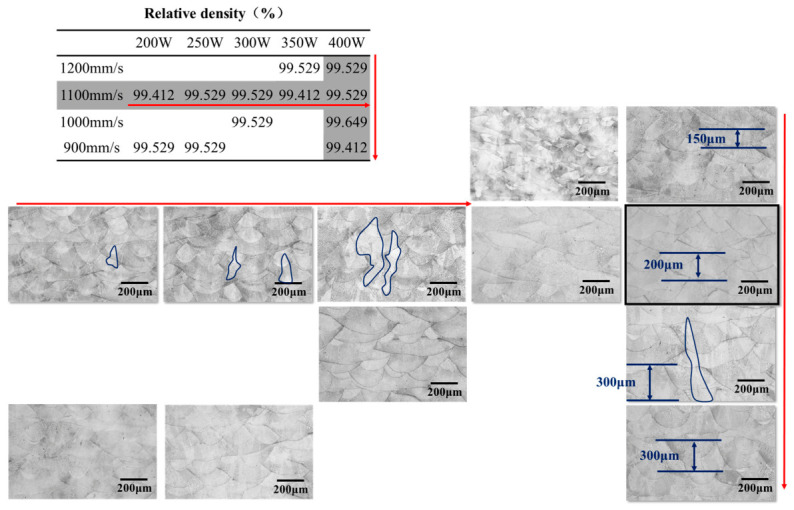
Melt pool morphology of as-deposited In625 specimens.

The melt-pool morphology of the as-deposited In625 samples was characterized to reveal the interaction mechanism between process parameters and microstructural evolution. Figure 5 presents the melt-pool morphology of the In625 samples, including those with excellent densification (the green samples in Figure 5) as well as several samples with relatively poor density. It was found that, with increasing laser power, the tendency of columnar grains to grow along the build direction became more pronounced. This is because the increase in laser power raised the energy input into the melt pool, thereby increasing the degree of undercooling in the liquid metal. According to solidification theory, greater undercooling promotes grain growth. To minimize the system’s free energy, columnar grains preferentially grow along the direction of the maximum temperature gradient during additive manufacturing [14,15].

However, when the laser power was further increased beyond 350 W, the stronger melt-pool convection caused by the high power made the temperature distribution within the melt pool more uniform. As a result, the temperature gradient along the build direction was no longer the dominant factor governing grain growth, and the size of the columnar grains no longer increased further. In addition, the larger energy input intensified evaporation and spattering within the melt pool, thereby disrupting the growth environment of the columnar grains, and the traces of their directional growth became less distinct. At a constant laser power, as the scanning speed decreased, the interaction time per unit area of material increased, allowing the material to absorb more energy. Consequently, the melt-pool depth gradually increased from 150 μm to 300 μm. When the scanning speed was reduced further, below 1000 mm/s, the melt-pool depth no longer increased. This is because the heat dissipation rate from the melt-pool surface to the surrounding environment reached a balance with the heat input from the laser. Overall, the sample fabricated at a laser power of 400 W and a scanning speed of 1100 mm/s exhibited a relatively stable melt-pool morphology. Combined with its high relative density and melt-pool stability, these parameters can be identified as optimal for additive manufacturing.

### 3.3. Effects of Heat Treatment on Microstructure, Defects, and Mechanical Properties

#### 3.3.1. Microstructural Evolution During Heat Treatment

**Figure 6:**
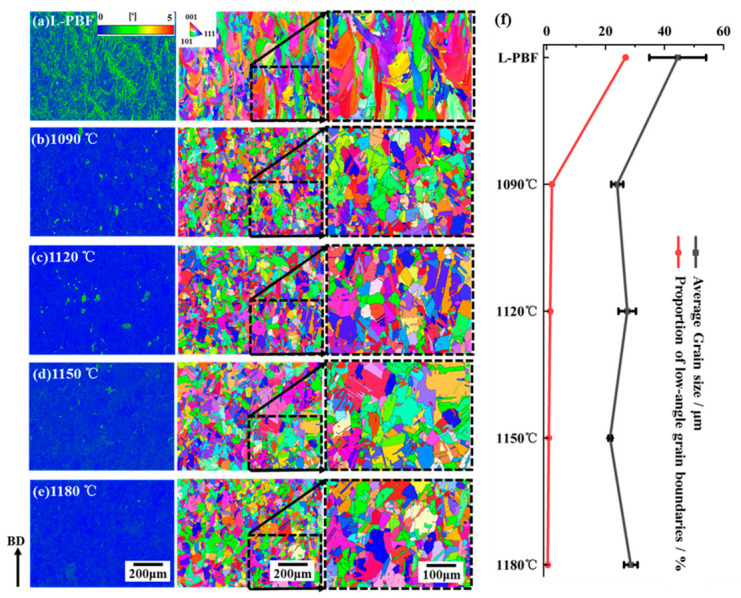
EBSD-based microstructural characterization of specimens before and after heat treatment, including grain morphology, orientation features, and kernel average misorientation (KAM) distributions. (**a**) As-built L-PBF specimen; (**b**) HT-1090 °C; (**c**) HT-1120 °C; (**d**) HT-1150 °C; (**e**) HT-1180 °C; (**f**) Average grain size evolution diagram.

The microstructures of the samples before and after heat treatment are shown in Figure 6. The surface microstructures parallel to the build direction were characterized for the as-deposited sample and for samples treated at different heat-treatment temperatures. A comparative analysis of KAM (kernel average misorientation) maps showed that the as-deposited sample exhibited the greatest lattice distortion. Lattice distortion reflects the extent to which atoms deviate from their ideal lattice positions in the crystal structure. The rapid solidification in L-PBF leads to compositional inhomogeneity within the melt pool.

Meanwhile, as shown in the EBSD images, the grain orientations in the sample were highly random. Under these conditions, atomic arrangements at grain boundaries must accommodate lattices with different orientations on both sides, resulting in extensive distortion concentrated at the grain boundaries in the as-deposited sample [16,17]. After heat treatment, the degree of lattice distortion decreased markedly, and the influence of temperature on lattice distortion was generally consistent among the heat-treated samples. In the present study, KAM and low-angle grain boundary fraction are used as semi-quantitative indicators of stored strain and dislocation-related lattice distortion, rather than as direct measurements of dislocation density. This indicates that heat treatment consumed the stored energy accumulated in the as-deposited samples owing to cyclic thermal effects. The large amount of stored energy introduced during L-PBF promoted the redistribution of solute atoms within the solvent lattice. It facilitated defect motions such as dislocation cross-slip and glide at elevated temperatures. As a consequence, lattice distortion was substantially reduced after heat treatment, while coarse grains gradually evolved into finer grains. The reduction in lattice defects and the formation of fine grains enhanced the material’s ability to accommodate grain-boundary sliding deformation, thereby improving the toughness of the heat-treated samples [18].

The degree of lattice distortion is closely related to the fraction of low-angle grain boundaries (2–10°). Lattice distortion promotes the multiplication and accumulation of dislocations, thereby providing favorable conditions for the formation of low-angle grain boundaries. Therefore, to some extent, the fraction of low-angle grain boundaries can be used to represent the extent of dislocation slip and lattice distortion in the material [19]. As shown in Figure 6e, the fraction of low-angle grain boundaries was quantified for samples in different conditions. After heat treatment, this fraction decreased significantly, and it continued to decline with increasing temperature. This is because, during heat treatment, dislocations within low-angle grain boundaries were rearranged through mechanisms such as climb, thereby reducing the grain-boundary energy [20,21]. As the temperature increased, dislocation motion became more active, further promoting grain growth. As low-angle grain boundaries are low-energy interfaces, they were gradually consumed by growing grains, resulting in a continuous decrease in their fraction from 1.86% at 1090 °C to 0.44% at 1180 °C. This suggests that a further increase in heat-treatment temperature contributes only limitedly to grain-boundary sliding in the material.

Figure 6 also presents the statistical results for grain-size distribution and average grain size before and after heat treatment. As shown in Figure 6a, the as-deposited sample exhibited a columnar grain morphology with an aspect ratio greater than 2. During additive manufacturing, the large temperature gradient promoted the growth of columnar grains along the deposition direction, with an average grain size of 44.5 μm. After heat treatment, the average grain size of all samples decreased significantly, and the microstructure changed to an almost fully equiaxed morphology containing a large number of twins. The appearance of numerous twins after heat treatment is likely associated with the increased grain-boundary mobility during recrystallization and grain-boundary migration, and reflects the microstructural adjustment toward a lower-energy configuration at elevated temperatures. The prolonged exposure to high temperature during heat treatment provided sufficient time for atomic diffusion and grain-boundary migration. As a result, the original non-equiaxed grains transformed into equiaxed grains to reduce grain-boundary energy, thereby producing a more stable grain morphology [22]. This also indicates that the equiaxed transformation temperature of L-PBF-fabricated In625 is lower than 1090 °C. The average equiaxed grain size ranged from 21.6 to 28.6 μm, with only slight differences among the heat-treated samples. However, subsequent analysis revealed significant differences in the mechanical properties of the materials treated at different temperatures. Based on the grain-size distribution, the relatively limited variation in average grain size among the heat-treated samples suggests that grain size alone may not fully account for the observed differences in mechanical properties.

#### 3.3.2. Defect Evolution During Heat Treatment

**Figure 7:**
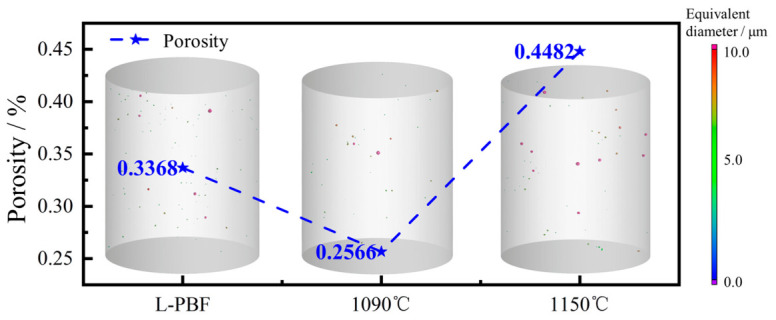
Three-dimensional defect distributions of the as-built and heat-treated specimens characterized by industrial CT, showing the change in pore population and porosity after heat treatment under different conditions.

Industrial CT was performed on samples in three different conditions after heat treatment to investigate the influence of key heat-treatment parameters on defect evolution. As shown in Figure 7, the porosity differed between the as-deposited sample and the heat-treated samples at 1090 °C and 1150 °C. The porosity of the as-deposited sample was 0.3368%. After heat treatment at 1090 °C for 1 h, some pores closed under the high-temperature condition, leading to a decrease in porosity to 0.2566%. At this stage, the reduction in porosity is interpreted mainly as the partial closure or shrinkage of small pores under enhanced atomic diffusion and local stress relaxation. The pores that closed at this stage were mainly small micropores. When the heat-treatment temperature was increased to 1150 °C, the porosity rose to 0.4482%, and the pore size increased markedly. This change is detrimental to the material’s strength. The growth of pores and other defects at elevated temperatures is more likely associated with stress redistribution induced by dislocation rearrangement, grain-boundary migration, and recrystallization-related microstructural evolution, rather than with direct pore generation by dislocation motion itself.

Meanwhile, during the equiaxed transformation, the stress distribution around internal pores changed, promoting the tearing and enlargement of some pores [23]. In addition, the increase in porosity may be associated with the growth of pores previously smaller than the detection limit (1 μm). Therefore, the observed porosity increase at 1150 °C should be interpreted as a combined result of pore coarsening/enlargement and the emergence of previously sub-resolution pores into the detectable range, rather than unequivocal formation of entirely new defects. Although the porosity increased after heat treatment, the material density remained above 99.5%, indicating that the densification level was still relatively high.

## 4. Discussion

### 4.1. Mechanism of Heat Treatment on the Microstructure of In625

**Figure 8:**
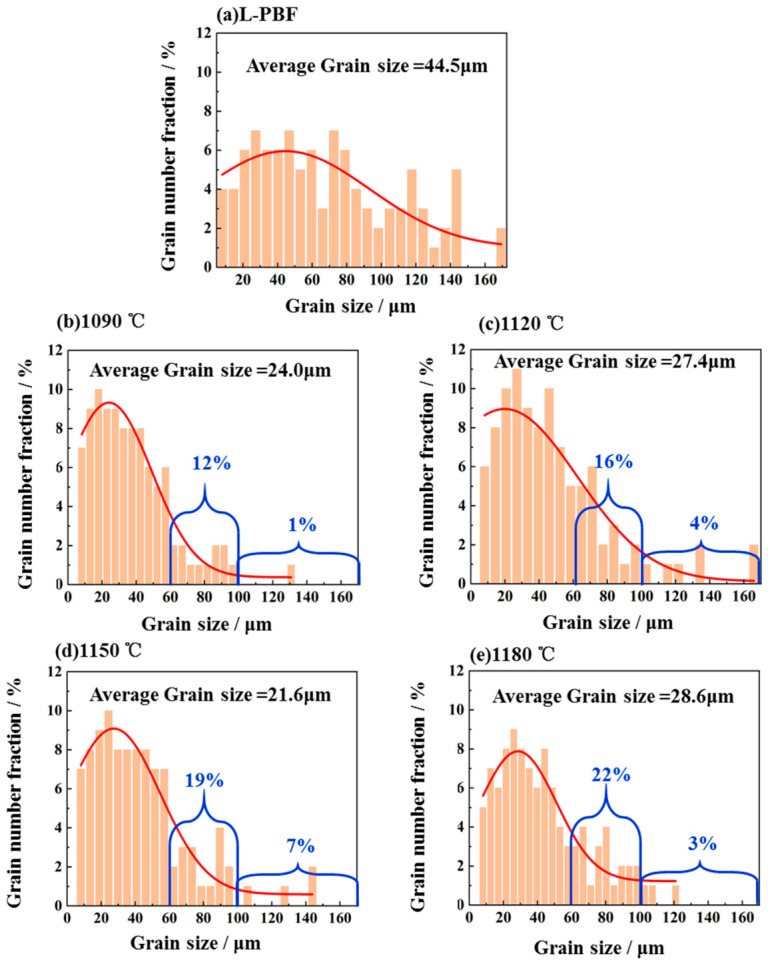
Grain size distribution of In625 under different conditions.

A quantitative statistical analysis of the grain size was conducted for samples before and after heat treatment. The statistical dataset was extracted from Figure 6, and the corresponding results are presented in Figure 8. Prior to heat treatment, the grain size was uniformly distributed between 0 and 160 μm. Grains exceeding 100 μm were predominantly elongated columnar structures. After heat treatment, the majority of grains in all samples were ≤100 μm in size, which can be attributed to the equiaxed transformation occurring at elevated temperatures. Notably, as indicated by the latter segment of the red Gaussian fitting curve in Figure 8, the proportion of grains larger than 100 μm gradually increased with rising heat-treatment temperature, followed by a decrease beyond 1150 °C. The fraction increased from 1% at 1090 °C to 7% at 1150 °C, attributed to enhanced grain growth at high temperature, where larger grains increasingly consumed adjacent smaller grains after the equiaxed transformation. However, the fraction decreased to 3% after treatment at 1180 °C. This reduction may be associated with the increased grain boundary mobility at higher temperatures, which can induce the formation of new grain boundaries within larger grains, thereby triggering secondary recrystallization [24]. Accordingly, the grain-size evolution after heat treatment should be understood as the combined result of an initial equiaxed transformation/recrystallization process and subsequent temperature-assisted grain growth, rather than as a simple monotonic coarsening behavior. It is also noteworthy that the proportion of grains in the 60–100 μm range increased continuously with increasing temperature, from 12% to 22%. This behavior can be explained by the sustained growth of grains within this size interval through the absorption of smaller grains, while their growth beyond the 100 μm threshold remained limited. It can therefore be inferred that at 1180 °C and higher temperatures, the fraction of grains in this size range may continue to increase, leading to a further rise in the average grain size.

**Figure 9:**
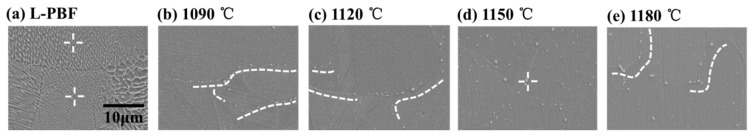
Distribution of precipitates in specimens before and after heat treatment.

**Figure 10:**
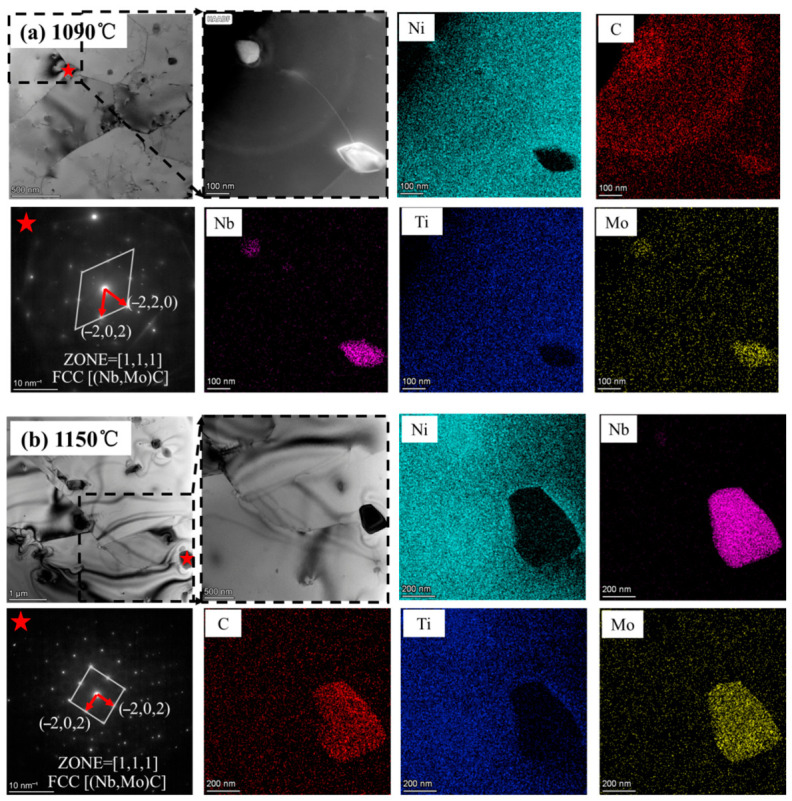
Elemental composition of specimens in different states determined by TEM.

**Figure 11:**
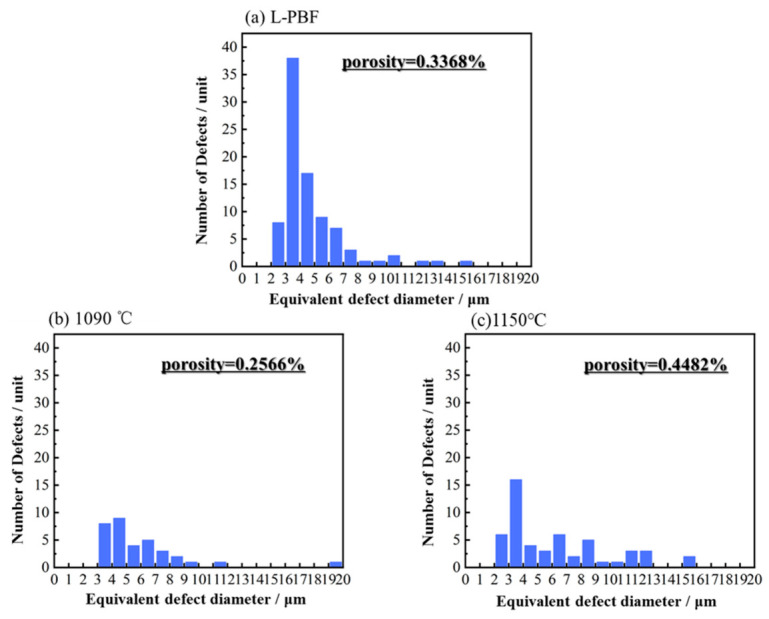
Defect size statistics of specimens in different states.

EDS elemental mapping was performed on samples before and after heat treatment. As shown in Figure 9, the white markers indicate the locations of precipitates. Before heat treatment, precipitates were distributed both along the melt pool boundaries and within the melt pool interior. Transmission electron microscopy (TEM) characterization was conducted on the as-deposited sample and heat-treated samples (1090 °C and 1150 °C) to clarify the compositional evolution and size variation in segregated phases with increasing temperature. The corresponding results are presented in Figure 10. In the as-deposited state, the segregated phases were primarily enriched in Nb, C, Ti, and Mo. After heat treatment at both temperatures, the precipitates were mainly enriched in Nb, Mo, and C, indicating that Ti dissolved into the matrix at elevated temperatures. This redistribution of alloying elements suggests that heat treatment affects not only carbide morphology but also the local solid-solution state of the matrix, which may further influence the balance between strength and ductility. The Ti initially precipitated in the as-deposited condition was fully re-dissolved into the matrix. As shown in Figure 11, diffraction analysis confirmed that the precipitated phases consisted of FCC-structured NbC and MoC. In the heat-treated samples, the segregated phases were mainly MC-type and M_6_C-type carbides. The MC-type carbides were Nb-rich and directly precipitated from the liquid phase, whereas the M_6_C-type carbides were Mo-rich and could also form at higher temperature ranges [25,26]. At 1090 °C, the precipitates exhibited elongated morphologies with sizes of approximately 100 nm or below. When the temperature increased to 1150 °C, the precipitate size increased to about 300 nm. This coarsening behavior can be attributed to enhanced atomic diffusion and Ostwald ripening at elevated temperatures, in which smaller precipitates dissolve and reprecipitate onto larger ones, leading to the gradual growth of the latter [27]. Such precipitate coarsening may reduce the uniformity of precipitation strengthening and increase local strain incompatibility between the matrix and coarse carbides, thereby promoting strain localization and increasing the likelihood of microcrack initiation during tensile deformation. Overall, within the temperature range of 1090–1150 °C, the heat-treatment temperature effectively regulates the size of precipitated carbides, while the chemical composition of the precipitates remains essentially unchanged.

### 4.2. Mechanism of Heat Treatment on Defects in In625

A quantitative statistical analysis of defect size distribution based on industrial CT was further conducted, and the results are presented in Figure 11. For both pre- and post-heat-treated samples, most defects were concentrated in the size range of 2–8 μm, with only a small fraction exceeding 10 μm. Smaller defect sizes are generally beneficial to ductility, as they reduce the likelihood of crack initiation during tensile deformation [28,29,30]. In the as-built L-PBF condition, the number of defects within the 2–8 μm range was significantly higher than that in the heat-treated samples. This suggests that heat treatment effectively reduces pores and related defects generated during deposition. A comparison between the 1090 °C and 1150 °C heat-treated samples revealed an increase in the number of defects within the 2–5 μm range, indicating that small defects tended to coarsen at higher temperatures. In addition, the 1150 –treated sample exhibited a higher number of defects exceeding 10 μm, resulting in an increase in porosity from 0.2566% to 0.4482%. This phenomenon may be attributed to intensified dislocation motion at elevated solution-treatment temperatures, accompanied by grain growth that weakens grain boundary cohesion. During the subsequent rapid argon-cooling process, partial tearing or expansion of existing pores and microcracks may occur, thereby altering the size and number of internal defects after heat treatment [31].

### 4.3. Effect of Heat Treatment Parameters on Mechanical Properties

**Figure 12:**
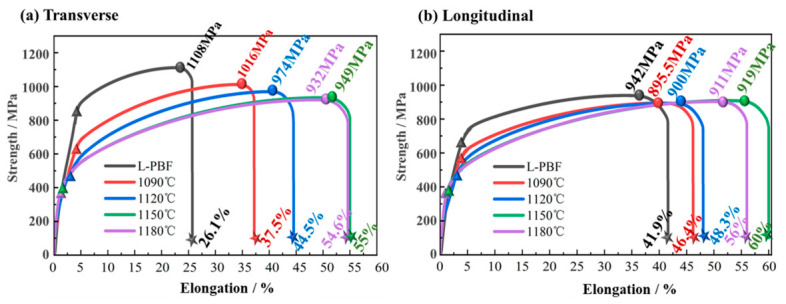
Room-temperature tensile properties of In625 under different conditions. (**a**) Test results of transverse specimens; (**b**) Test results of longitudinal specimens.

As shown in Figure 12, the room-temperature mechanical properties of In625 under different conditions were evaluated. Specimens fabricated parallel to the additive manufacturing deposition direction were denoted “longitudinal,” whereas those fabricated perpendicular to the deposition direction were denoted “transverse.” The letters and numbers represent samples subjected to different heat-treatment temperatures. As illustrated in Figure 12a, specimens heat-treated to 1150 °C showed a significant improvement in toughness. However, when the temperature increased to 1180 °C, toughness decreased in both the longitudinal and transverse directions. This behavior is primarily attributed to abnormal grain growth at elevated temperatures (see Figure 8e), which leads to coarse grains that reduce the material’s capacity for plastic deformation [32]. In addition, comparison of tensile curves with identical color coding indicates that the degree of strength anisotropy gradually weakened with increasing heat-treatment temperature, as evidenced by the convergence of ultimate tensile strength peaks represented by the same color. The directional differences in toughness were 15.8%, 8.9%, 3.8%, 5%, and 1.7%, respectively, showing an overall decreasing trend. A slight rebound was observed at 1150 °C, which may be attributed to partially recrystallized regions within the microstructure at this temperature, potentially leading to a temporary increase in anisotropy in some specimens. This slight rebound may also be associated with local microstructural heterogeneity, including non-uniform precipitate evolution or local grain-growth differences, and should therefore be regarded as a plausible interpretation rather than a definitive conclusion. The mechanical testing results confirm that elevated temperatures effectively promote grain equiaxation. The consumption of low-angle grain boundaries and the progression of dynamic recrystallization facilitate the transformation of grain morphology toward a more equiaxed structure. The enhancement of equiaxation is of significant importance for the subsequent service performance of the material.

The tensile curves, tensile strength, and elongation data of specimens fabricated along different orientations were compared, as shown in Figure 12. A decreasing tendency in the tensile strength of the transverse specimens was observed with increasing heat-treatment temperature. However, when the temperature increased from 1150 °C to 1180 °C, the decline in strength markedly slowed, with the difference in ultimate tensile strength between the two temperatures remaining within 20 MPa. This indicates that temperatures above 1150 °C do not significantly enhance elongation. Considering that higher temperatures also led to increased porosity and more pronounced defect coarsening, the selection of heat-treatment temperature should be understood as a trade-off among ductility, strength retention, and defect stability. For the longitudinal specimens, the effect of heat-treatment temperature on ultimate tensile strength was relatively minor, with variations in less than 25 MPa across the four temperatures.

In contrast, the elongation of longitudinal specimens was significantly affected. This behavior can be attributed to the fact that, in the as-built condition, most columnar grains in longitudinal specimens grow along the build direction. Elevated heat-treatment temperatures promote the transformation of columnar grains into equiaxed grains, thereby improving the coordination of plastic deformation during tensile loading. Meanwhile, the reduced low-angle grain boundary fraction and the alleviation of lattice distortion after heat treatment are also consistent with the improved ductility, although the present study does not establish a strict one-to-one quantitative correlation among these parameters. Moreover, higher heat-treatment temperatures facilitate the dissolution of elements into the matrix, reducing the pinning effect of precipitates during furnace cooling and thereby enhancing toughness. Notably, when the heat-treatment temperature reached 1150 °C or higher, both strength and elongation declined. Microstructural analysis revealed that the fraction of coarse grains increased within this temperature range. The reduction in total grain boundary area facilitated dislocation motion along preferred directions, thereby diminishing the material’s resistance to deformation [33,34]. Therefore, within the investigated heat-treatment window, 1150 °C provided the most favorable overall balance among ductility improvement, acceptable strength retention, and anisotropy reduction.

It should be noted that the present study considered a fixed holding time of 1 h and four discrete solution-treatment temperatures, while air cooling was adopted as the only post-treatment cooling route. In addition, the CT-based defect analysis was limited by the voxel resolution (~1 μm), which means that sub-resolution pores could not be directly resolved. Therefore, the detailed transition mechanisms may be further refined in future work by examining intermediate temperatures, alternative time–temperature combinations, different cooling strategies, and higher-resolution defect characterization. Moreover, although the present work focused on room-temperature tensile behavior, the observed evolution of grain morphology, pore characteristics, and carbide precipitation may also be relevant to high-temperature service properties such as creep, fatigue, and oxidation resistance. Residual stress relaxation was inferred indirectly from the reduction in lattice distortion and the heat-treatment process, but no direct residual-stress measurement was performed in the present study.

## 5. Conclusions

This study systematically investigated the effects of laser powder bed fusion (L-PBF) processing parameters and post-solution heat treatment on the microstructure, defect evolution, and mechanical properties of In625. Based on the experimental results and mechanistic analysis, the following conclusions can be drawn:

(1) An optimal L-PBF processing window was identified for fabricating high-density In625 components. A laser power of 400 W, combined with a scanning speed of 1100 mm/s, produced specimens with relative densities exceeding 99.5% and a stable melt pool morphology.
(2) Solution heat treatment effectively promoted grain equiaxation and reduced lattice distortion in L-PBF-fabricated In625. Nearly complete grain equiaxation was observed after heat treatment at 1090 °C and above under the present experimental conditions, with average grain sizes remaining below 100 μm, while higher temperatures led to progressive grain coarsening associated with enhanced grain-boundary migration.
(3) Defect evolution during heat treatment exhibited a non-monotonic trend. Heat treatment at 1090 °C facilitated partial closure of small pores, reducing porosity from 0.33% to 0.25%. In contrast, higher temperatures (≥1150 °C) were associated with pore enlargement/coarsening and an increase in porosity to approximately 0.45%, which may involve stress redistribution during grain-boundary migration together with the growth of previously sub-resolution pores into the CT-detectable range.
(4) Heat treatment reduced mechanical anisotropy and enhanced ductility. Within the investigated heat treatment range, the samples treated at 1150 °C exhibited a comparatively favorable combination of strength, ductility, and anisotropy reduction, with tensile strength exceeding 919 MPa and elongation approaching 60%. However, considering that the mechanical properties obtained at 1190 °C were also very close, a definitive determination of the optimal heat treatment condition would require further statistical validation.

## Figures and Tables

**Table 1:** Chemical composition of major alloying elements in In625 powder.

Elements	Ni	Fe	Mn	C	Si	Cr	Al	Ti	Nb + Ta	Mo	P
w/%	62	2.23	0.01	0.06	0.08	22.01	0.23	0.14	3.89	9.32	0.01

**Table 2:** Variables of the additive manufacturing process parameters.

Power (W)	Scanning Speed (mm/s)	Volume Energy Density (J/mm^3^)
200	900/1000/1100/1200	33.3~44.4
250	900/1000/1100/1200	41.7~55.6
300	900/1000/1100/1200	50.0~66.6
350	900/1000/1100/1200	58.3~77.8
400	900/1000/1100/1200	66.6~88.9

## Data Availability

The data presented in this study are available in the article. Further inquiries can be directed to the corresponding author.

## Abbreviations

The following abbreviations are used in this manuscript:

L-PBF	Laser Powder Bed Fusion
IPF	Inverse Pole Figure
KAM	Kernel Average Misorientation
CT	Computed Tomography
AM	Additive Manufacturing
In625	Inconel 625
